# Antimalarial drug discovery – the path towards eradication

**DOI:** 10.1017/S0031182013000826

**Published:** 2013-07-17

**Authors:** JEREMY N BURROWS, EMILIE BURLOT, BRICE CAMPO, STEPHANIE CHERBUIN, SARAH JEANNERET, DIDIER LEROY, THOMAS SPANGENBERG, DAVID WATERSON, TIMOTHY NC WELLS, PAUL WILLIS

**Affiliations:** Medicines for Malaria Venture, ICC, 20 Route de Pré-Bois – PO Box 1826, 1215 Geneva 15, Switzerland

**Keywords:** malaria, *Plasmodium*, eradication, Target Product Profile, MMV

## Abstract

Malaria is a disease that still affects a significant proportion of the global human population. Whilst advances have been made in lowering the numbers of cases and deaths, it is clear that a strategy based solely on disease control year on year, without reducing transmission and ultimately eradicating the parasite, is unsustainable. This article highlights the current mainstay treatments alongside a selection of emerging new clinical molecules from the portfolio of Medicines for Malaria Venture (MMV) and our partners. In each case, the key highlights from each research phase are described to demonstrate how these new potential medicines were discovered. Given the increased focus of the community on eradicating the disease, the strategy for next generation combination medicines that will provide such potential is explained.

## INTRODUCTION

Malaria is a parasitic infection of the genus *Plasmodium* and has historically been one of the deadliest diseases for humankind. In recent years, there has been a reduction in the numbers of deaths from malaria, and this has been attributed to the use of insecticide-treated bed-nets, indoor residual spraying to kill mosquitoes (the vector that carries the parasite and infects humans) and the availability of effective, well tolerated and high quality medicines (Roll Back Malaria, 2012). Indeed according to the WHO there were 655 000 deaths in 2010 which is a substantial reduction when compared with over a million at the turn of the millennium (WHO, 2011).

However, since a strategy focused on case management risks is unsustainable in the long term there has been an increased focus to target the elimination and eradication of the parasite (Alonso *et al.* 2011). This challenging goal will require the combined and sustained application of the entire community including: (1) renewed efforts to develop insecticides that overcome known resistance pathways and kill all mosquitoes; (2) the delivery of effective vaccines that protect infants and children; (3) the delivery of appropriate and sensitive diagnostics to guide health care tactics, and (4) the discovery, development and delivery of new medicines that not only clear the asexual blood stage parasites and cure patients, but also kill the asymptomatic and vector-stage forms that allow transmission of *Plasmodium* (Burrows *et al.* 2011*a*.)

It is clear that the fight against malaria will involve chemotherapy as a vital component (White *et al.* 2011). However, resistance to the existing medicines is always a risk. Consequently, all treatments must be combinations of two or more active ingredients such that no compound is exposed as a monotherapy to high levels of parasites for a significant period of time wherever possible (Duparc *et al.* 2012).

## CURRENT FIRST LINE THERAPIES

### Treatment of clinical symptoms of malaria

The vast majority of cases of malaria are uncomplicated disease, where the patient has fever and up to 200 000 parasites per microlitre. Artemisinin combination therapies (ACTs), the first line medicines in this case, are extremely safe and effective after three days of dosing. These are medicines that combine an artemisinin derivative (for example artemether, artesunate or dihydroartemisinin) with a partner drug. Artemisinin is a natural product and is extracted from the leaves of the *Artemisia annua* plant. Artemisinin derivatives are semi-synthetic peroxides and are active against all species of *Plasmodium*; they are able to clear blood-stage parasites and reduce fever rapidly (WHO, 2010). Combining an artemisinin derivative with a partner drug in one tablet (a fixed-dose combination) is preferred, as it ensures that both active ingredients are taken, maximizing efficacy and reducing the potential for resistance developing.

**Fig. 1.**
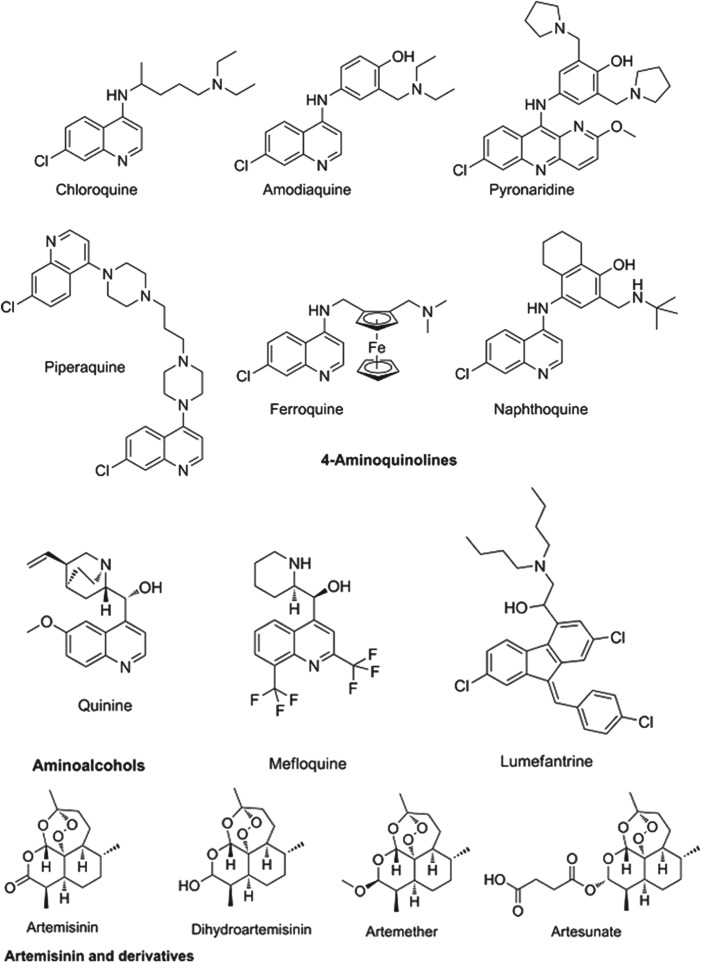
Structures of clinical antimalarials for asexual blood stages.

The partner drugs that are used in these combinations also share their origins in nature, being synthetic variants on the natural product quinine. Broadly speaking they separate into two chemical classes: the amino-alcohols, which share the closest similarity to quinine, and the 4-aminoquinolines, which were early synthetic mimics. The amino-alcohol class is best illustrated by mefloquine and the more structurally divergent lumefantrine. Chloroquine, the original exemplar of the 4-aminoquinoline class is no longer in use for *falciparum* malaria due to reduced parasite sensitivity. It is now replaced by more recent analogues without cross resistance: amodiaquine, piperaquine and the ‘quinacrine-amodiaquine’ hybrid, pyronaridine. Structures of these molecules and other clinical derivatives of each class are shown in Fig. 1 (Burrows *et al*. 2011).

### Treatment of liver-stage vivax malaria

**Fig. 2.**
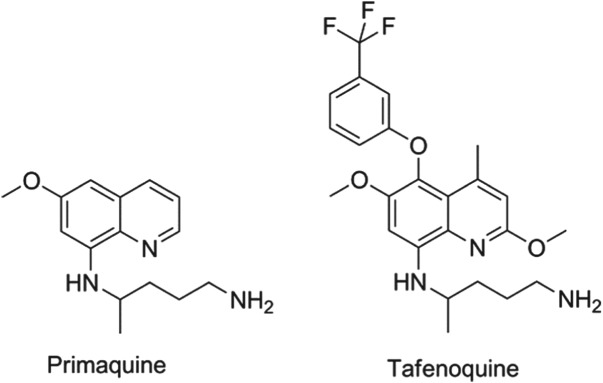
Structures of clinical antimalarials for vivax radical cure.

In all cases of infection, the parasite passes from the mosquito to the liver, usually via the regional lymph nodes. For *Plasmodium falciparum*, the dividing or liver schizont stage lasts for around 6 days. However, for *P. vivax* (the key species in Asia and the Americas) and *P. ovale* there is also a sub-population of the liver-stage parasites, called hypnozoites, that appears to halt its development and become dormant, only to relapse after a period which can vary between four weeks and several years. Once the primary blood-stage infection has been treated, the patient is asymptomatic and often unaware of the risk associated with retention of these parasites. Although the biological mechanisms underlying the activation process are not understood, it is clear that hypnozoites can ‘wake up’ and reactivate leading to a new blood-stage infection in the absence of a mosquito bite – a relapse (Price *et al.* 2007). There is one drug only that is approved for relapse prevention and that is the 8-aminoquinoline, primaquine. Primaquine, however, is a 14-day treatment that is given to patients who are effectively asymptomatic, but it has known gastro-intestinal side effects and, more importantly, presents a risk of haemolytic anaemia to patients who have low activity of the glucose 6-phosphate dehydrogenase and hence termed ‘G6PD deficient’ (Baird and Rieckmann, 2003). Consequently, new molecules with improved oral dosing regimens and no-G6PD deficiency liability are a priority (Wells *et al.* 2010). The communities’ immediate hope in this regard is the 8-aminoquinoline, tafenoquine, which benefits from being a single dose. Tafenoquine is undergoing Phase II/III studies with GSK and MMV with the aim of establishing the minimum efficacious and safe dose. The structures of primaquine and tafenoquine are shown in Fig. 2.

### Transmission blocking

Another way to eliminate the disease is to break the transmission cycle by preventing patients infecting mosquitoes (Sinden, 2009). The transmission-blocking potential of many of the existing mainstay treatments is still poorly understood (Delves *et al.* 2012). This is partly due to the complexity of clearly measuring an impact on clinical transmission (i.e. from human to human) but also because the biological area, spanning parasite stages in the host and mosquito, is extremely intricate and the necessary tools are only now starting to emerge (Sinden *et al.* 2012*a*, *b*). It is clear, however, that a single dose of primaquine of 0·75 mg/kg is effective as a gametocytocidal agent. As such, it clears mature stage gametocytes (often called stage V), the infectious parasite form to the mosquito, from the blood of patients. Indeed, WHO recommend this in combination with ACTs as a way of killing all the gametocytes in patients, preventing the development of mosquito-stage parasites and thus, by inference, blocking transmission. However, this dose level has restrictions: the risk of haemolysis due to G6PD deficiency should be taken into account and the drug should not be given to pregnant women or infants under 1 year of age. In 2012, after a review of all data, WHO extended their recommendation further to include lower single dose primaquine of 0·25 mg/kg for all confirmed malaria patients in elimination regions where the higher single dose primaquine is not already in use. This low dose is still not recommended for pregnant women or infants but is deemed ‘unlikely to cause serious toxicity in subjects with any of the G6PD variants’ (WHO, 2012*a*, *b*).

### Chemoprotection

**Fig. 3.**
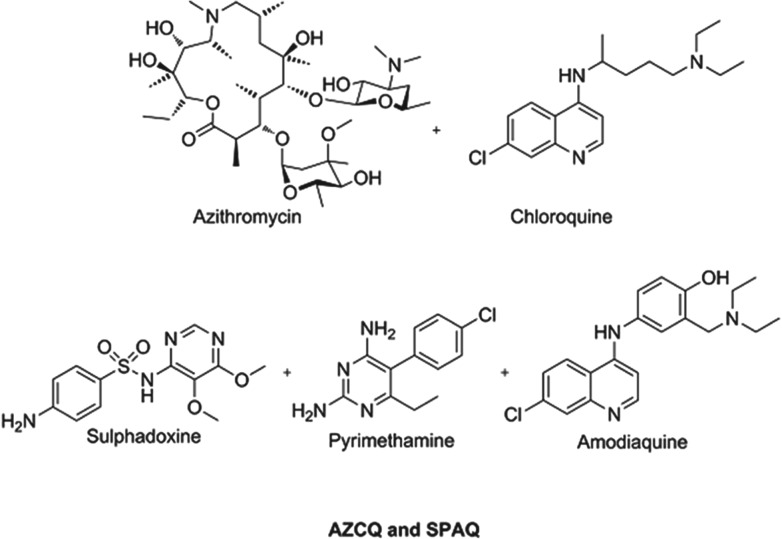
Structures of clinical combination chemoprotectants.

Preventing malaria in vulnerable populations, particularly expectant mothers and young children, is still a priority, especially given that the development of fully protective vaccines is a very challenging task for protozoal infections. Given the vulnerability of this patient sector, and the fact that they do not have malaria at the time of dosing, it is imperative that such medicines are extremely safe and well tolerated. Consequently, the combinations being profiled or in use today comprise historical antimalarials which have an extremely well understood safety profile and, despite field resistance, are combined in a way to deliver benefit. A combination of azithromycin and chloroquine (AZCQ) is being developed for presumptive treatment and future chemoprotection in pregnancy (Chico *et al.* 2008). The combination shows synergy, in that it is active in populations infected with chloroquine-resistant strains. For infants, recent field data show the success of a triple combination of sulphadoxine–pyrimethamine–amodiaquine (SPAQ) in protecting children in the Sahel region of sub-Saharan Africa, where the season is only four months long (WHO, 2012*a*, *b*). Because these medicines are historical they are relatively inexpensive and the cost to protect a child for a year will be between US$0·40–0·60, making it extremely cost effective. Structures of these chemoprotectants are shown in Fig. 3.

## RESEARCH PRIORITIES FOR DEVELOPING NEW DRUGS FOR ERADICATION

Over the past decade, ACTs have played a pivotal role in malaria control programmes. These drugs will continue to play an important role in the journey towards eradication as they will remain the cornerstone of case management. ACTs are extremely well tolerated and efficacious, but there are areas for improvement in the next generation of medicines. Artemisinin and partner drug resistance is under close scrutiny; ideally, in combination, two drugs would have similar pharmacologically effective durations in patients, so as to protect each other comprehensively – this is not achieved in an ACT; finally, for eradication, a combination that is extremely well tolerated across a population and that clears all parasites in an individual is desirable. Thus, there is a critical need for a new generation of medicines to target the challenges of the eradication agenda (The malERA Consultative Group on Drugs, 2011). Two main types of medicines are envisioned, one to treat and the other to protect, and Target Product Profiles (TPPs) have been developed that outline the requirements for these new medicines. (1) TPP1 – A combination of blood-stage cure, with transmission blocking, hypnozoiticidal and post-treatment prophylactic action, which matches or improves on the simplicity of the ACT 3 day regimen and (2) TPP2 – A single-exposure chemoprotection, which matches or improves on the current weekly chemoprotection given by mefloquine, but without the adverse gastrointestinal or CNS events.

With safe and efficacious products already on the market, delivery of a multiple-dose blood-stage treatment will only be relevant if mainstay therapies fail due to resistance. Patients have been characterized in the Thai - Cambodian border region who show a significantly slower response to artesunate in terms of parasite clearance and, unless this can be contained, there will be parts of the world in the near future where ACTs are ineffective (Noedl *et al.* 2008; Phyo *et al.* 2012). Another potential differentiation for a new medicine would be the potential to be administered as a single oral dose, which could be observed directly. This would eliminate the possibility that an incomplete course of therapy is given, and incomplete therapeutic courses of anti-infectives are one of the prime causes for the spread of resistant strains.

An ideal combination requires a variety of activities: blood-stage cure, transmission blocking, anti-relapse and post-treatment prophylactic action; this is the Single Exposure Radical Cure and Prophylaxis combination (SERCaP) as highlighted from the output of the malERA consortium and is the first Target Product Profile – TPP1. Such a medicine would transform drug treatment for malaria.

A second type of medicine needed in the early stages of elimination is that of chemoprotection, and is described by TPP2. Again, ideally this Single Exposure Chemoprotectant (SEC) would be able to be given on a once per month basis with the knowledge that between dosing periods individuals were protected from primary blood-stage infections.

As discussed earlier, any new medicines developed should be a combination of at least two complementary molecules. Following consultations with its partners, MMV has defined four Target Candidate Profiles (TCPs), which aim to guide the identification of compounds that might be combined to produce a medicine that meets the TPPs described above (Burrows *et al.* 2013). The four TCPs are: (1) TCP1 – Rapid parasite clearance molecules that could replace artemisinin; (2) TCP2 – Molecules with long half-lives to give post-treatment prophylaxis, and replace the current generation of aminoquinolines or aminoalcohols; (3) TCP3 – Molecules that kill non-dividing parasite forms such as hypnozoites to stop *P. vivax* relapse (termed 3a) and gametocytes to stop transmission (termed 3b); and (4) TCP4 – Slow-onset chemoprophylactics or causal prophylactics with long duration and excellent safety profiles to protect vulnerable populations in once endemic regions.

## GLOBAL MALARIA PORTFOLIO

Over the last decade there has been an increased investment in antimalarial research and development and through the work of organizations such as Medicines for Malaria Venture (MMV), their partners and others, new molecules with novel modes of action are entering into pre-clinical development and beyond (Anthony *et al.* 2012). The current Global Malaria Portfolio can be found at the following link: http://www.mmv.org/research-development/rd-portfolio and this is updated on a quarterly basis.

Despite the relative abundance of projects at certain stages, taking account of attrition between each phase and the need for combination medicines, it is clear that a sustained delivery of high quality antimalarial preclinical candidates is necessary for at least a decade.

## MMV PORTFOLIO

Within the MMV portfolio, two new compounds have recently completed Phase IIa studies and there are an additional six compounds in Phase I or Preclinical Development. A selection of these is described below.

### OZ439

The most advanced of the new molecules is a second generation endoperoxide, OZ439, that has been designed to have superior pharmacokinetics to the artemisinins. OZ439 has demonstrated clinical efficacy as a single agent (in phase IIa studies, the so-called proof of concept). It is now being tested in combination safety studies, and will start combination efficacy studies in 2014. Studies in both healthy volunteers and infected patients show a significant plasma exposure for as long as 20 days, suggesting it might be possible to use as part of a single-dose therapy for uncomplicated malaria (Möhrle *et al.* 2012). In patients, OZ439 drives the reduction of parasites at about the same rate as artesunate. The next step is to identify a suitable combination partner that will give long-term protection and support a single-dose cure.

**Fig. 4.**
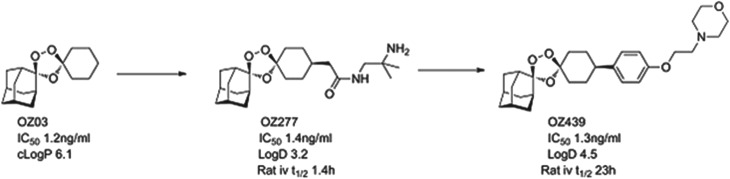
OZ439: Hit to Candidate.

OZ439 comes from the ozonide class of antimalarials and was delivered by a project team led by Jonathan Vennerstrom at Nebraska University, in collaboration with the Swiss Tropical and Public Health Institute (Swiss TPH), Monash University and MMV (Charman *et al.* 2011). Vennerstrom and co-workers hypothesized that the endoperoxide in an ozonide could deliver efficacy equal to that observed with the natural product artemisinins. The team demonstrated that a stabilized ozonide, OZ03, with a simple structure was sufficient to give excellent *in vitro* potency. The high lipophilicity and low solubility, however, had to be addressed and this led to an introduction of polarity and ionizable groups in a region of the molecule that was synthetically tractable yet which did not compromise potency. This initially led to OZ277 (arterolane) which was the first clinical candidate and which was licensed to Ranbaxy (Vennerstrom *et al.* 2004). Indeed the combination of OZ277 and piperaquine received approval in India in 2012 under the name Synriam™, and has been widely used to treat malaria patients in India in the last six months. OZ277, however, has lower exposure in patients than expected and this was hypothesized as being due to instability in infected blood, due to an interaction with ferrous iron. Vennerstrom's team, therefore, focused on replacing the amide with a phenyl ring, that helped to stabilize the ozonide, and they substituted the amide for an ether-linked base. The resultant compound, OZ439, was shown to have improved infected blood stability, was able to cure mice infected with *P. berghei* from a single 30 mg/kg dose and was progressed as the potential single dose cure candidate (see Fig. 4).

### NITD609

A second compound in Phase II is a novel, synthetic spiroindolone, developed by the Novartis Institute for Tropical Diseases (NITD) in Singapore in a collaboration that included the Swiss TPH, the Dutch Biomedical Primate Research Center, the Genomics Institute of the Novartis Research Foundation (GNF) and MMV. It was the first compound to be developed using whole-cell screening against the parasite (Rottmann *et al.* 2010), and started human proof of concept in 2012, just under 5 years after the initial screen began. It has now passed proof of concept, and has been shown to clear parasites in patients infected with either *P. vivax* or *P. falciparum*. The compound inhibits the P-type sodium transporter ATPase 4 (PfATP4), and this results in an increase in the concentration of sodium ions in the parasite, which is toxic to the cell.

**Fig. 5.**
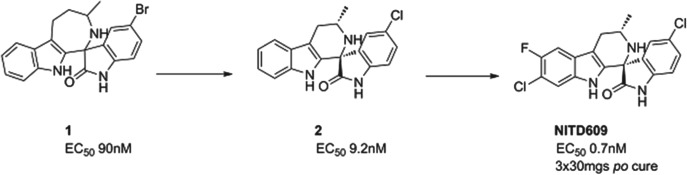
NITD609: Hit to Candidate.

The spiroindolone class was found from screening a library of natural product and ‘natural product like’ compounds at Novartis. The starting ‘hit’, compound **1**, had an intriguing structure, good potency and, impressively for a starting point, suppressed parasitaemia in the *P. berghei* mouse model of malaria by >99% with a single dose of 100 mg/kg. Excellent medicinal chemistry was applied to contract the seven-membered ring, to define the stereochemical structure/activity relationship and to replace the lipophilic bromine atom. This resulted in compound **2** which had an increased potency yet reduced lipophilicity – the ideal outcome from medicinal chemistry. However, its metabolic stability was still non-optimal; this was fixed by judicious positioning of halogens on the tetrahydro-beta-carboline ring. The resulting compound had even greater potency and excellent pharmacokinetics – NITD609 (Yeung *et al.* 2010; see also Smith *et al.* in this Special Issue of Parasitology – volume 140, 2013) see Fig. 5.

### GNF156

A second molecule from the Novartis consortium, led this time from the Genomics Institute of the Novartis Research Foundation (GNF) in San Diego, delivered GNF156 as a new clinical compound, again in collaboration with MMV. The compound, an imidazolopiperazine, has high potency against blood and liver stages *in vitro* and *in vivo*, and has been shown to kill gametocytes demonstrating potential in transmission blocking. The series acts *via* a novel mechanism, involving a previously unannotated gene now called *P. falciparum* Cyclic Amine Resistance Locus (Pfcarl) (Meister *et al.* 2011). The compound is well tolerated in preclinical safety studies, and has now safely completed phase I studies.

**Fig. 6.**
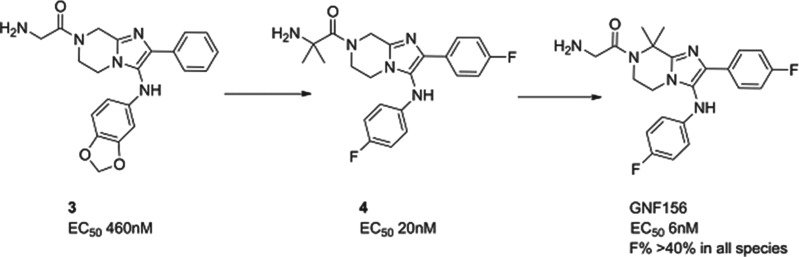
GNF156: Hit to Candidate.

The imidazolopiperazine series, exemplified by **3**, was also identified from whole cell screening. The first task was to replace metabolically labile aromatic substituents and to stabilize likely positions of metabolism with blocking halogens. This led to compound **4** whereby potency and stability had both been improved – though not sufficiently for a candidate. Metabolite identification and excellent medicinal chemistry then led to the isomer, GNF156, whereby the metabolically susceptible position on the piperazine was blocked with two methyl groups. GNF156 demonstrated a good overall profile with an ED_99_ in the *P. berghei* mouse of 1·1 mg/kg (Nagle *et al.* 2012) as shown in Fig. 6.

### DSM265

**Fig. 7.**
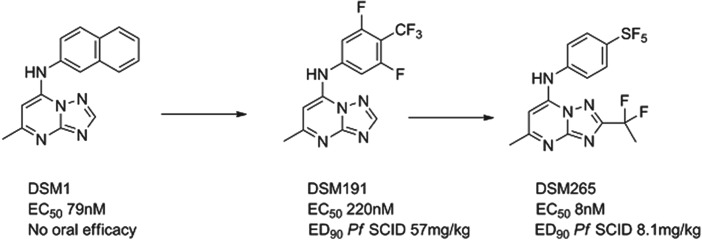
DSM265: Hit to Candidate.

The *P. falciparum* enzyme dihydroorotate dehydrogenase (*Pf*DHODH) is known to be essential for the survival of the parasite. A team led by Meg Phillips at the University of Texas Southwestern, in collaboration with the University of Washington, Monash University and MMV identified a potent and selective triazolopyrimidine-based inhibitor DSM1 *via* a high-throughput enzyme screen. The three-dimensional structure of the enzyme-inhibitor complex was resolved and the subsequent lead optimization programme led to the identification of the preclinical candidate. DSM1 was an interesting molecule but with non-optimal pharmacokinetics on repeat dosing and insufficient potency. First of all, progress was made to improve pharmacokinetics by substituting electron-withdrawing groups in the aniline ring; due to the hydrophobic nature of the binding site only lipophilic groups have had major success here, e.g. DSM191 (Gujjar *et al.* 2009). A second breakthrough came using the X-ray crystal structure; it was clear that limited substitution off the triazolo carbon could be achieved and, combined with electron withdrawal could reduce desolvation of the heterocycle and improve potency. These changes, Fig. 7, in collaboration with GSK, delivered DSM265, which has a good potency and safety profile from preclinical studies (Coteron *et al.* 2011). If successful, it will be the first antimalarial chemotherapy to target DHODH. The compound is currently in preclinical development, with the expectation to enter Phase I trials in early 2013.

### P218

**Fig. 8.**
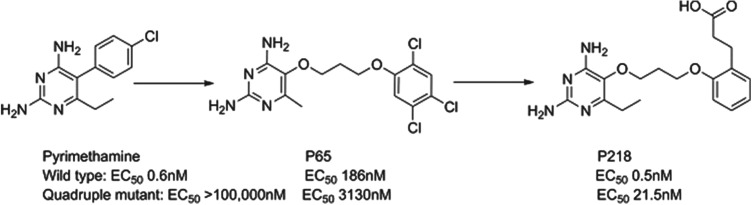
P218: Pyrimethamine to Candidate.

Dihydrofolate reductase (DHFR) inhibitors such as pyrimethamine have been widely used for the treatment of malaria, although their clinical efficacy has been compromised by resistance. P218 is a next generation inhibitor of DHFR that has been delivered by the Thai BIOTEC group in collaboration with Monash University, the London School of Hygiene and Tropical Medicine and MMV; it has largely finished preclinical development. Impressively, the team designed P218 using structure-based design methods to have a high affinity to both the parent and mutated enzymes and to also kill both wild type and clinically-relevant resistant strains. The challenge of sustaining activity across even quadruple-mutated enzymes was overcome by starting with the pyrimethamine scaffold and then using a flexible side-chain that could adopt a variety of conformations to achieve potency such as P65. A carboxylic acid was also found to be optimal in selective binding to a key protein arginine in the parasite, but not in man (see Fig. 8). P218 has a good pharmacokinetic profile, is selective and highly efficacious; the initial safety testing with P218 indicated a good safety margin between the toxicity in animals and the predicted effective human dose (Yuthavong *et al.* 2012).

## KEY CRITERIA FOR NEW TREATMENTS FOR MALARIA

In the development of new treatments for malaria a number of key generic criteria need to be met by any drug candidates, in addition to those eradication criteria already discussed: high potency, low cost, high stability in tropical conditions, good development potential (e.g. acceptable solubility), a low chance for resistance developing, and high levels of safety in the target populations.

The development of resistance is a constant challenge in the treatment of malaria as with any infectious disease. In the development of new medicines it is important that molecules are selected that have a low potential for resistance development. This means screening against existing targets and resistance mechanisms and selecting molecules that, ideally, hit more than one target to increase resilience to the development of resistance. The frequency of resistance development and the fitness cost to the parasite of resistance are other factors that should be taken into account. Resistant phenotypes that have a high fitness cost would require extreme selective pressure to spread. A standardized *in vitro* method to assess these characteristics quantitatively in *P. falciparum* during the early phases of the drug development process has recently been developed (Ding *et al*. 2012). Notably, assessing the risk of resistance induction has helped project teams to down-prioritize series, allowing them to focus their efforts on more promising candidates (Sanz *et al.* 2011).

Finally, a high level of safety is of paramount importance. The populations most vulnerable to malaria at this time are pregnant women and children under 5 years of age. Chemoprevention is already a key malaria control strategy in these populations and as malaria prevalence decreases according to an elimination scenario, the wider population at large also become vulnerable due to decreased immunity. Consequently, many of the individuals that will receive the new chemoprotective agents will not have clinical malaria. This raises the bar in terms of the levels of safety required for any new medication.

### Discovery of molecules to clear blood-stage parasites: TCP1 – rapid clearance; TCP2 – long duration

The rapid increase in the availability of genome data and high-throughput screening technology over the past decade initially raised hopes for the identification and development of many new classes of drugs for malaria (Burrows and Waterson, 2011). For some time it was thought that understanding the malaria parasite at the molecular level would accelerate the discovery of new antimalarials by identifying compounds that could inhibit key enzymes or cell receptors in the parasite. Unfortunately, these molecular-based approaches have not proved efficient in identifying validated targets (Payne *et al.* 2007).

For parasitic diseases such as malaria, screening can also be carried out against the whole cell, and this has proved an extremely valuable approach. A considerable advantage of parasite-based screening is that it can identify compounds that act on more than one molecular target. Drugs that attack more than one cellular pathway should be less vulnerable to the development of resistance (Ding *et al*. 2012).

The industrial partners of the malaria research community have been quick to adopt this alternative approach and by leveraging their ability to generate automated systems, pharmaceutical companies and institutes such as Eskitis under the leadership of Avery and colleagues have screened over six million compounds against the erythrocytic stages of the malaria parasite (Duffy and Avery, 2012; Guiguemde *et al.* 2012). This has led to the identification of over 25 000 compounds that show some effect on the parasite at sub-micromolar concentrations. Data on many of these compounds has been released to the malaria community online to encourage drug-lead identification efforts. These data can be located at: https://www.ebi.ac.uk/chembl/malaria/. GlaxoSmithKline (Gamo *et al.* 2010), Novartis (Plouffe *et al.* 2008) and St. Jude Children's Research Hospital (Guiguemde *et al.* 2010) have released chemical structures and associated information on approximately 19 000 compounds with confirmed blood-stage activity against *P. falciparum* that were identified from a combined screening effort of over 4 million compounds from the companies’ and University's chemical libraries. In December 2011, MMV announced that it was making a library of 400 compounds with confirmed activity against the blood-stage of *P. falciparum* available to the research community free of charge. Each of the compounds was selected, pragmatically on availability and other criteria, from the published data sets listed above (Spangenberg *et al.* 2013).

**Fig. 9.**
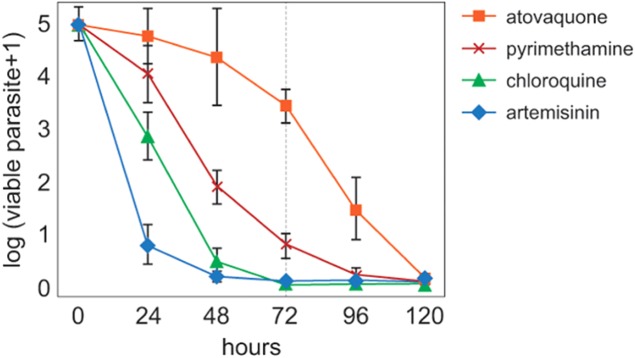
The Parasite Reduction Ratio for four standard antimalarials: artemisinin, chloroquine, pyrimethamine and atovaquone. Reproduced from ©Sanz LM *et al*. PlosONE 7(2) e30949. doi:10.1371/journal.pone.0030949

The widespread adoption of whole-parasite screening has led to the generation of a new assay ‘the parasite reduction ratio’ that allows the direct determination of *in vitro* killing rates for potential antimalarials (Sanz *et al.* 2012). A number of *in vitro* techniques have been developed to investigate the effect of drug treatment on asexual parasite survival and growth, and are used for both drug development and resistance-monitoring studies. Although these assays use a variety of approaches, they all rely on measuring metabolic activity as a proxy for parasite viability. However, relying on proxies can lead to incorrect interpretations. For example, viable but metabolically inactive parasites could be measured as dead, while parasites committed to death could still display metabolic activity. The new assay is based on limiting serial dilution of treated parasites and re-growth monitoring which allows the direct *in vitro* measurement of the effect of antimalarial compounds on parasite viability. In addition, drug lag phase, that is the time required for a drug to achieve its maximal killing, can be precisely identified and timed. The parasite reduction ratio clearly shows the different speeds of action of atovaquone, pyrimethamine, chloroquine and artemisinin (Fig. 9). This is particularly relevant with respect to finding molecules that are deliberately fast (TCP1) or slow onset (TCP4) blood-stage killers.

The parasite clearance rates of potential antimalarials can also be measured in two *in vivo* systems, which are complementary to the parasite reduction ratio. The first system examines the impact of the candidate molecule on *P. berghei* in mice. However, the use of a rodent plasmodial species as a surrogate for *P. falciparum* is a drawback of this model. The second system uses a *falciparum* murine model of malaria by generating strains of *P. falciparum* that can infect immunodeficient mice engrafted with human erythrocytes – the humanized SCID mouse (Angulo-Barturen *et al.* 2008; Jimenez-Diaz *et al.* 2009).

Despite the focus of screening currently on whole-cell systems, it does not mean that target-based drug discovery is redundant. The lack of target-based drug discovery projects is more a result of the lack of high quality targets than the approach itself. Indeed, if whole cell screening hits are discovered with attractive phenotypes, then target-finding techniques can be highly relevant as a strategy to identify a back-up series from a target-based screen.

Finally, finding compounds with long durations of exposure in patient blood sufficient to maintain efficacy for periods of time beyond just curing the patient , hence also protecting against new infections (TCP2), is also a priority. Pharmacokinetics clearly becomes the defining parameter in this case and once *in vitro* metabolism has been significantly reduced, there is likely to be need for substantial preclinical *in vivo* pharmacokinetic work. Following this subsequent modelling to humans, demonstration of predicted clinical relevance is needed, as well as extensive safety testing to ensure an acceptable cardiovascular risk in addition to demonstrating that a prolonged exposure is well tolerated. In addition, early *in vivo* screening of whole cell actives can be an efficient method to identify starting points rapidly for further optimization.

### Developing new drugs for the treatment of *P. vivax:* TCP3a – relapse prevention

The lack of safe and easily administered drugs for radical cure of *P. vivax* is a critical gap in the malaria eradication agenda. To achieve eradication, the entire human reservoir of malaria parasites must be eliminated, including latent dormant stages. This will be particularly difficult to achieve for *P. vivax* because of the relapsing liver-stage hypnozoites that are refractory to treatment with all current antimalarials except primaquine.

One of the key challenges in developing new drugs for *P. vivax* is the inability to conduct cellular screens for anti-relapse agents. To develop a *P. vivax* liver-stage assay, the malaria community needs to secure a supply of *via*ble sporozoites. Maintaining *P. vivax* in continuous *in vitro* culture to provide a clonal supply of parasites on which the mosquitoes can feed is the major barrier to this. Stable and infective hepatocyte lines are also needed, as primary human liver cells are often of variable quality and lose their differentiation in culture (Wells *et al.* 2010).

A standard cascade for the discovery and development of drugs for anti-relapse with *P. vivax* would start with a simple cell assay, followed by confirmation of the activity *in vivo* in an animal model, and then proceed *via* clinical proof of concept in an ‘Out of Transmission’ model to full human clinical trials. With no cell assay for *P. vivax*, the historical foundation of anti-relapse drug discovery has been use of an *in vivo* system: the infection of rhesus monkeys with *P. cynomolgi*. This simian parasite relapses in a regular time frame (around 22 days) and thus new compounds can be dosed once the patent blood stages have been cleared and the animal parasitaemia monitored for a relapse. It was this model that was originally used in the screening that led to the discovery of the drug primaquine and the follow-on compound, tafenoquine. However, for the development of a large drug discovery programme an *in vitro* model is required to facilitate high-throughput screening of compounds. Cultured hepatic stages of *P. cynomolgi* have been established in rhesus hepatocytes but determination of which non-dividing forms correspond to hypnozoites has proven difficult. It has been suggested that, by noting the response of these non-dividing forms to an anti-relapse drug and an antimalarial that is not active against hypnozoites, a better characterization of true hypnozoites can be obtained (Dembele *et al*. 2011). It is this *in vitro* assay that is currently the pragmatic primary assay for anti-relapse data to drive medicinal chemistry.

Thus a key priority is to deliver a liver-stage assay of *P. vivax* to drive future medicinal chemistry in the pursuit of new anti-relapse medicines.

### Focusing on transmission blocking: TCP3b – gametocytocidal agents

As mentioned above, primaquine is effective in blocking transmission to the mosquito from a single human dose of 0·75 mg/kg. WHO has also recommended use of a lower dose (0·25 mg/kg) under certain circumstances and this is therefore the gold standard to beat. The challenge will persist whilst this recommendation remains (and that will depend on the results of future studies), until extensive resistance to primaquine is demonstrated or, finally, hitherto unknown adverse events from the low dose become apparent. However, the unsuitability for pregnant women and very young children highlights improvements necessary in next generation molecules.

The strategy is to target host gametocytes such that mature gametocytes are either killed or rendered inactive. In such a scenario, following a mosquito bite parasite gamete formation is blocked either because of an absence of mature gametocytes in the blood, or their inactivation. With gamete formation inhibited in the mosquito then there cannot be further development of vector-stage parasites and hence the life-cycle is blocked. This approach reduces the transmission window for a patient and the length of this time-window is defined by the rate with which gametocytes are killed or inactivated. For primaquine this transmission window appears to be around 24–48 h (Rieckmann *et al.* 1968).

A second type of activity of a compound can be direct on the mosquito stages of the parasite. Such activity is desirable, in that it will result in transmission being blocked even during the transmission window. The risk is that in the absence of a gametocytocidal agent such a transmission window can be very long and hence, unless a drug has a phenomenally long half-life, for as long as mature gametocytes circulate, its effectiveness is likely to be limited (Bousema *et al.* 2010).

Pragmatically, the current priority is to develop molecules that combine host gametocytocidal activity of an equal potency to that displayed against asexual blood stages. The optimum cascade for identifying compounds affecting transmission involves an assay that confirms inhibition of mature gametocytes followed by a standard *falciparum* membrane feeding assay (SMFA). Potential additional *in vivo* work including a mouse-to-mouse transmission may also be instructive particularly in order to compare against primaquine, which requires metabolism for efficacy (Sinden *et al.* 2012*a*, *b*).

### Chemoprotection for malaria elimination: TCP4 – prophylactics

In a malaria-elimination scenario, individuals will be at risk of reinfection from areas that retain a human reservoir of parasites. In this context, drugs will be needed for chemoprotection of individuals in once endemic areas. To prevent chemoprotection strategies from jeopardizing drugs used for the treatment of clinical disease through the development of resistance, it is important that the two types of antimalarial are well differentiated. One possible mechanism for facilitating this could be to examine the potential of slow-onset blood stage-acting molecules (such as those with a delayed death phenotype) or those that only kill liver schizonts for chemoprotection strategies.

Longer duration blood-stage assays (96 h) can be run and compared with results from earlier time points (48 h or 72 h) to prioritize compounds with slow blood-stage action (Dahl and Rosenthal, 2007). To identify series that kill the developing liver stages, high-throughput screening can be performed against rodent liver-stage parasites in human liver cell lines (e.g. HepG2) (Meister *et al.* 2011). *Falciparum* and *vivax* liver-stage assays are still embryonic but, ideally, it would always be important to confirm activity on the human parasite prior to significant investment in a drug discovery programme.

*In vivo* rodent models are available such as the *P. berghei* sporozoite infection model and these can even be performed using imaging to detect parasitaemia prior to a blood stage infection (e.g. Sanchez *et al.* 2004).

## SUMMARY

Currently, the mainstay treatments for malaria are effective and safe. Furthermore, new clinical entities that offer significant advances over existing regimens continue to progress well and a range of compound and project options is available in Preclinical Development and earlier. However, the tenacity of the parasite in thwarting our attempts to kill it is unparalleled. If we are serious about controlling and ultimately eradicating the parasite then together we need sustained efforts to discover, develop and deliver new medicines that not only cure patients, but block transmission, block relapse and offer protection against new infections. The advent of the new MMV TPPs and TCPs will help to guide researchers towards these ends.
